# Forty hyperbaric oxygen therapy sessions for traumatic brain injury: “why?”

**DOI:** 10.4103/mgr.MEDGASRES-D-25-00134

**Published:** 2026-01-27

**Authors:** Paul G. Harch

**Affiliations:** Section of Emergency Medicine, Department of Medicine, Louisiana State University Health Sciences Center, New Orleans, LA, USA

##  

Borlongan and Hadanny’s perspective on the rationale behind the use of 40 hyperbaric oxygen therapy (HBOT) sessions for patients with (chronic) traumatic brain injury (TBI) involved identifying mild TBI persistent postconcussion syndrome (PPCS) pathology as microvascular damage, citing the modern-era studies on HBOT in chronic mTBI PPCS which feature 40 HBOTs, and explaining the angiogenesis from 40 HBOTs in chronic wounds as a recapitulation of developmental plasticity.^1^ The actual “Why?” in this perspective was never explained, but is embedded in “How?” 40 HBOTs originated. This complementary perspective elucidates the “Why?” and “How?” 40 HBOTs have come to be used in mTBI PPCS. Simply, 40 HBOTs are provided for mTBI PPCS because it is the number of HBOTs: (1) that has historically been effective clinically and in research for treatment of chronic non-central nervous system (CNS) wounds, (2) that has historically been effective clinically for chronic CNS wounds, (3) the number demonstrated in human and animal research to produce angiogenesis in chronic wounds, and (4) the number that has now been validated in clinical trials of HBOT in mTBI PPCS.

HBOT is the modern-era form of hyperbaric therapy which uses >95% oxygen as the breathing gas instead of room air.^2^ HBOT is best understood as a treatment for acute, subacute, and chronic disease pathophysiology primarily in wounding conditions and infectious or inflammatory conditions.^3^ The number of treatments is dictated by the underlying pathology at the time of treatment, which is dependent on the time of intervention post insult, injury, or disease-onset. Wound pathology evolves from acute to chronic through inflammatory, proliferative, and remodeling phases.

HBOT delivered immediately after an ischemic, hypoxic, mechanical, chemical, or other injury is treating different pathologies than treatment delivered one week, one month, 6 months, or years after the insult.

**History of 40 HBOTs in chronic central nervous system wounding:** A single HBOT delivered within a few hours of insult has been the standard of care for Undersea and Hyperbaric Medicine’s signature diagnosis, acute decompression illness (DCI), and acute carbon monoxide poisoning.^3^ Reperfusion injury and decompressed gas (decompression illness) are the primary targets of immediate HBOT in these cases. As treatment is delayed tissue pathology evolves to ischemic/hypoxic wounds and the number of HBOTs increases to truncate the wound pathophysiology and stimulate repair, similar to treatment of subacute and chronic non-central nervous system wounds.^3^ This was demonstrated in divers in the 1980s and 1990s (**Figure 1**)^4^ where patients with many hours to months delay to treatment, or retreatment of incompletely treated neurological diving cases, required longer courses of treatment (40 or more) performed at an atypical lower pressure. The application of blocks of 40 HBOT treatments to significantly delayed diving cases derived from the 15-year experience of Dr. Richard A. Neubauer in the treatment of chronic CNS wounds in multiple sclerosis patients.^5^ Neubauer’s experience with multiple sclerosis patients and other adult and pediatric patients with chronic cerebral disorders was that patient’s receiving approximately 30-40 treatments sustained a measurable durable clinical benefit.^6^,^7^,^8^ Neubauer’s recommendation for 40 HBOTs was made to this author in the first application of HBOT to a homicidal patient with chronic decompression illness/dementia in 1990.^7^,^8^ The positive result with this diver was repeated in a second diver with the same delayed and retreated neurological DCI who improved with repetitive blocks of 40 HBOTs.^7^,^8^ Harch, Gottlieb, and Van Meter reported these cases and others with single-photon emission computed tomography (SPECT) brain imaging documentation at the annual Undersea and Hyperbaric Medical Society scientific meetings in 1992, 1993, and 1994, culminating in a report of 21 cerebral diving cases at the 1995 45^th^ UHMS Workshop on Treatment of Decompression Illness (**Figure 1**).^4^

**Figure 1:**
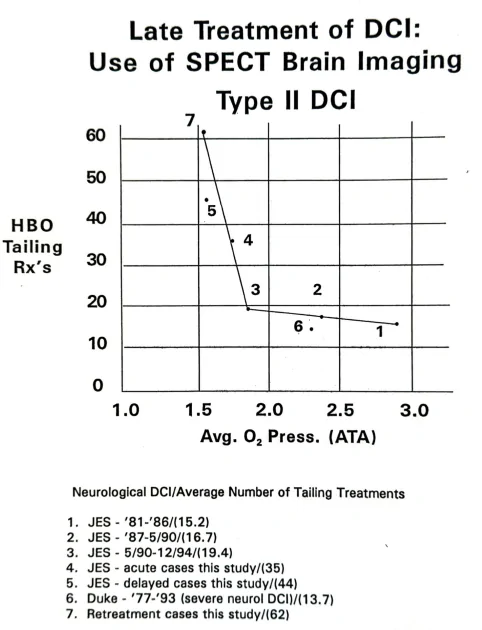
HBOT treatment number *versus* pressure for neurological decompression illness. Number of HBOT Tailing Treatments *versus* pressure of treatment of neurological decompression illness cases at JES, and Duke University Department of Hyperbaric Medicine.^4^ Permission for use of this figure from *Undersea & Hyperbaric Medical*, North Palm Beach, FL. ATA: Atmospheres absolute; DCI: decompression illness; HBO: hyperbaric oxygen; HBOT: hyperbaric oxygen therapy; JES: JoEllen Smith Medical Center New Orleans; SPECT: single-photon emission computed tomography.

**History of 40 HBOTs in chronic TBI:** Simultaneously with the HBOT treatment of divers with chronic DCI, Van Meter and Gottlieb applied extended courses of HBOT to boxers with dementia pugulistica.^7^,^8^ In 2010 Harch reported this study, the above chronology of single and repetitive blocks of 40 HBOTs for chronic DCI and other neurological conditions, and their extension to chronic TBI.^8^ In short the HBOT-induced positive clinical and SPECT findings in the Dementia Pugulistica study were duplicated by 40 and 80 HBOTs in the off-label application of HBOT to additional patients with chronic TBI and other chronic neurological conditions,^6^,^7^,^8^ replicated in an animal model of single and repetitive 40 HBOT blocks in chronic TBI,^9^ and further replicated in a community hospital based IRB with SPECT (1994-1999), the Perfusion Metabolism Encephalopathies Study, which evaluated single and repetitive blocks of 40 HBOTs for any chronic cerebral-based neurological condition.^7^,^8^ Harch and colleagues found that chronic TBI was the most responsive to 40 and 80 1.5 ATA HBOTs of all of the neurological conditions investigated.^7^,^8^ Harch and colleagues focused the investigation of 40 1.5 ATA HBOTs on a series of U.S. war veterans, presented the results at the Department of Defense Consensus Conference on HBOT in TBI, Washington, D.C., 12/5-6/2008, and investigated 40 HBOTs at 1.5 ATA in the LSU Pilot Trial of HBOT in mTBI/PPCS and PTSD.^6^ This 40 HBOT protocol was subsequently adopted in international and U.S. Department of Defense chronic TBI studies.^6^

The 40 HBOT experience in chronic DCI, TBI, and other CNS conditions inadvertently confirmed the 30-year practice-generated 40-HBOT application to the two chronic non-CNS wound conditions representing over 80% of clinical hyperbaric medicine in the U.S: diabetic foot wounds and chronic radiation wounds.^3^ The 40 HBOTs necessary to heal these non-CNS wounds were derived from practice habits and studies based on these practice habits (diabetic foot wounds) and the work of Dr. John Marx in radiation injury where 40 HBOTs delivered to a hypoxic, hypocellular, hypovascular radiation wound produced angiogenesis from 12 HBOTs through ~25 HBOTs.^10^ To investigate the possibility of angiogenesis in the brains of Dr. Neubauer’s patients, the boxers, divers, TBI, and other patients Harch and colleagues commenced a series of experiments in an animal model of mild and severe chronic TBI, applying 0, 40, and 80 HBOTs.^8^ Angiogenesis and improvement of lost spatial memory was greater at 80 HBOTs than 40. The benefit of 80 HBOTs was confirmed in a second study with sham air treatments and an altitude control group.^9^ These studies demonstrated angiogenesis and improved spatial memory at 40 and 80 HBOTs. The results were consistent with the early clinical experience in chronic TBI patients, however, 40 treatments was chosen to perform the TBI study^6^ due to replication of Dr. Neubauer’s original experience, the logistical and expense challenges of doing a 40 *versus* 80 treatment pilot study, and evidence of oxidative stress/toxicity at ~60 HBOTs in a group of 5 chronic CO poisoned patients presented at the Gulf Coast Chapter Meeting of the UHMS in 1995 (abstract available upon request).

**Rationale for 40 HBOTs in chronic TBI:** “Why provide 40 sessions of hyperbaric oxygen therapy to patients with traumatic brain injury?” Because it is the number of HBOTs established by habitual clinical application to achieve healing in chronic non-central nervous system wounding conditions (diabetic foot wounds and radiation injuries),^3^ achieve improvement in hundreds to thousands of patients with a variety of chronic neurological conditions,^4^,^5^,^6^,^7^,^8^ including cerebral palsy and patients with chronic TBI,^6^ the number validated in an animal model of chronic TBI,^9^ and the number now shown to be effective in adult chronic mTBI PPCS.^6^ Forty HBOTs, however, is not a ceiling number. In the original case series of severe TBI, stroke, divers, and other neurological patients, 80 HBOTs had a greater clinical effect similar to the animal TBI model.^6^,^7^,^8^,^9^ This seemed to be true for those patients with more severe injuries, but may also be dependent on the patient’s pathology, e.g., oxygen toxicity at 60–70 HBOTs in the carbon monoxide poisoned patients previously mentioned. The oxygen toxicity effect in these patients may have resulted from the use of 90-minute treatments at 1.5 ATA without air breaks. Sixty HBOTs now appear to be successful in a variety of studies performed by Drs. Hadanny and Efrati in Israel. However, eighty HBOTs are also showing benefit in the recent HBOT mTBI study by Weaver et al.,^11^ similar to the animal studies^8^,^9^ and clinical experience.^6^,^7^

**Mechanism of action of 40 HBOTs in chronic TBI:** The “How?’ that 40 treatments are achieving clinical improvement in patients with mTBI PPCS was not apparent until 2008. The HBOT experience in chronic non-CNS and CNS wound conditions suggests a fundamental property of wounded tissue, namely, a responsiveness to 40 intermittent exposures to elevated pressure and hyperoxia. Wound healing consists of new tissue growth. To grow new tissue, cells must be stimulated to divide and multiple. This requires DNA-signaling. DNA-signaling has been well-established as a mechanism of action of HBOT in 2008 and the signal appears to be the transient rise and fall of barometric pressure and oxygen.^6^ This mechanism of action appears to be distinctly different from the process in ontological vasculogenesis.^1^ The proposed explanation of HBOT-induced healing in mTBI PPCS is that HBOT is treating microscopic and macroscopic wounds in the brain.^6^ The presumption is that HBOT stimulates the growth of new vasculature in the presumed hypovascular wounds of mTBI. This is supported by the Marx HBOT osteoradionecrosis studies,^10^ the rat HBOT mTBI studies,^8^,^9^ and the imaging in HBOT mTBI studies,^6^ but it has not been established with the equally effective lower oxygen (hyperbaric air) doses of HBOT in the U.S. Department of Defense mTBI studies,^6^ and in the HBOT cerebral palsy studies. It remains to be proven that hyperbaric air can generate angiogenesis in a chronic brain wound.

In conclusion, this perspective on why 40 HBOTs should be delivered to patients with mTBI PPCS is that 40 HBOTs has been the number of intermittent exposures to increased barometric pressure and hyperoxia that has resulted in healing/improvement in chronic non-CNS and CNS wounding conditions through decades of clinical use and studies, the number that has been shown to generate angiogenesis in both animals and humans with chronic wounds, and the number that has now proven effective in mild TBI PPCS. It is the result of broad-based gene stimulation that is unique to hyperbaric/hyperbaric oxygen therapy.

*Dr. Harch is the owner of Harch Hyperbarics, Inc., an S-corporation, which is the vehicle for his private practice. No conflicts of interest exist between Harch Hyperbarics, Inc. and publication of this paper*.

*PGH is an Editorial Board member of Medical Gas Research. He was blinded from reviewing or making decisions on the manuscript. The article was subject to the journal’s standard procedures, with peer review handled independently of this Editorial Board member and his research groups*.

*The author declares that no generative AI was used in the preparation of this manuscript.*
